# Supplementary material for: Combined multidimensional single-cell protein and RNA profiling dissects the cellular and functional heterogeneity of thymic epithelial cells
Source: Nat Commun. 2023 Jul 10;14:4071. doi: 10.1038/s41467-023-39722-9 (PMC10333192; doi:10.1038/s41467-023-39722-9)
Supplement: Supplementary file 1 — Supplementary Information [file 41467_2023_39722_MOESM1_ESM.pdf]

Supplementary Figure 1

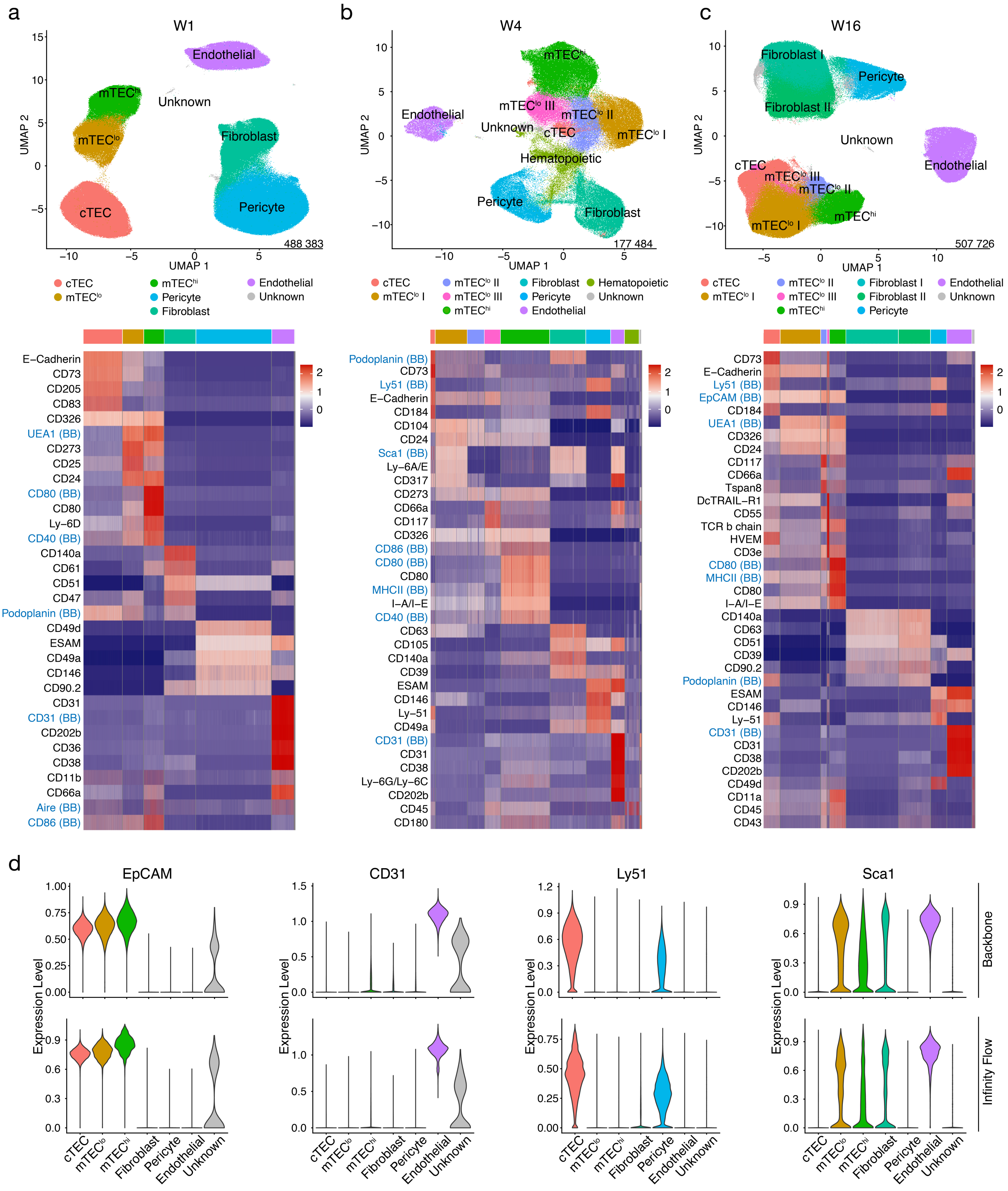

Supplementary Figure 1 | Infinity Flow analysis on thymic stromal cells

(a-c) Infinity Flow analysis was used to impute the expression of surface markers on $CD45^-$ cells derived from thymi of (a) 1- (n = 23), (b) 4- (n = 7), and (c) 16-week-old (n = 12) C57BL/6 WT mice, respectively. Hierarchical clustering analysis was performed on (a) 488383, (b) 177484, and (c) 507726 $CD45^-$ cells, respectively, and projected in a 2-dimensional space using UMAP (top panels; 7 to 10 clusters were obtained per timepoint). Each colour represents a specific cluster as indicated. Heatmaps (bottom panels) display the expression of the top 7 markers upregulated in each cluster (log fold-change > 0.2). Backbone (BB) markers have a blue font. **(d)** Violin plots comparing the expression of the indicated markers based on the backbone staining (top panels) and the prediction of Infinity Flow based on exploratory measurements of the same proteins (bottom panels).

Supplementary Figure 2

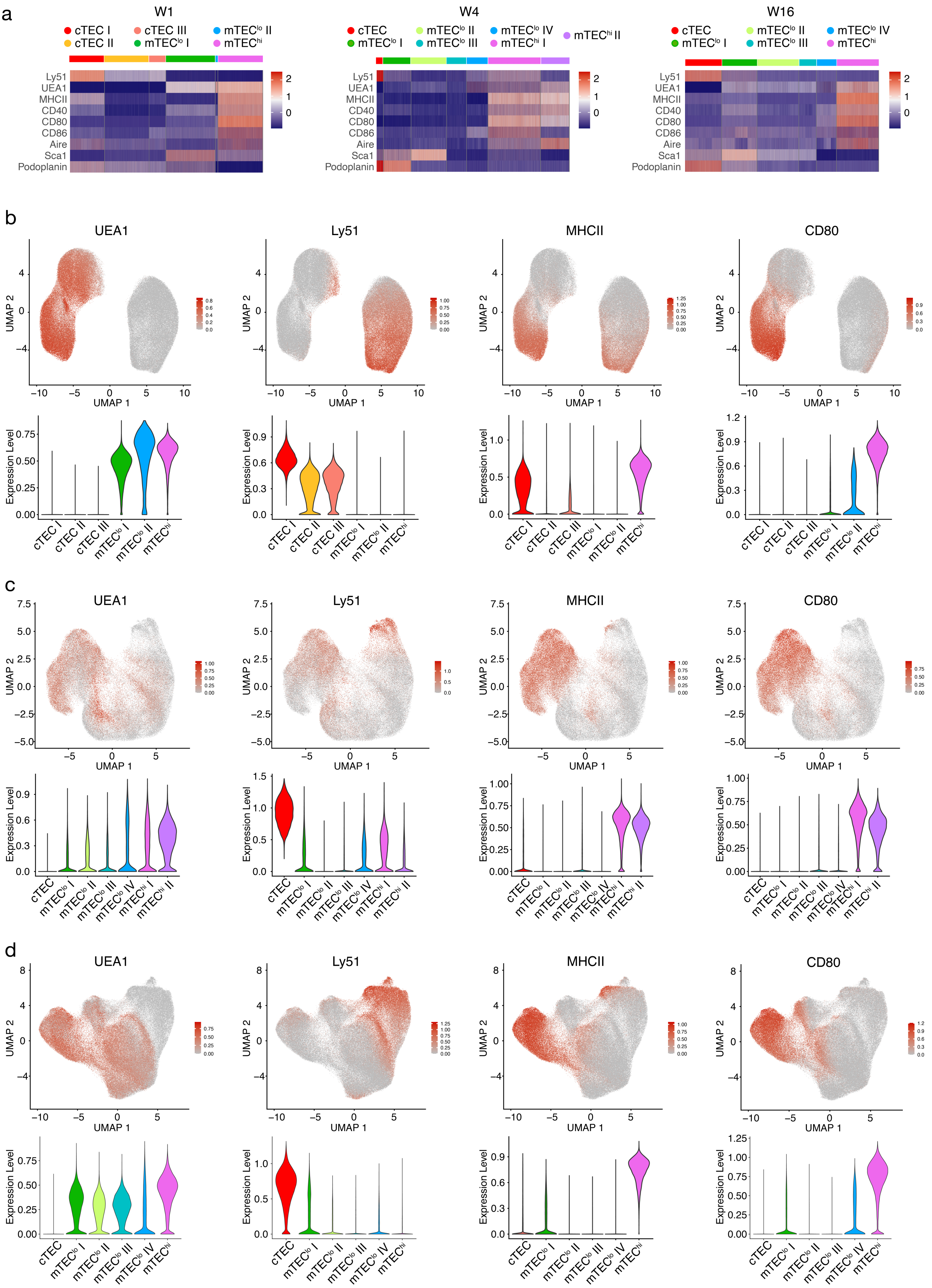

Supplementary Figure 2 | Classification of clusters based on UEA1, Ly51, MHCII, and CD80 expression
(a) Infinity Flow analysis was used to impute the expression of surface markers on TEC ($CD45^{-}EpCAM1^{+}$) derived from thymi of (b) 1- (n = 23), (c) 4- (n = 7), and (d) 16-week-old (n = 12) mice. Heatmaps display the expression of the backbone markers used for the LEGENDScreen. **(b-d)** UMAP graphs (top panels) and violin plots (bottom panels) illustrating the expression of UEA1, Ly51, MHCII, and CD80 on TEC from (a) 1-, (b) 4-, and (c) 16-week-old mice. Colour gradient indicates expression levels in the UMAP graphs and colours in the violin plots represent the different clusters, as defined in Figure 1b.

Supplementary Figure 3

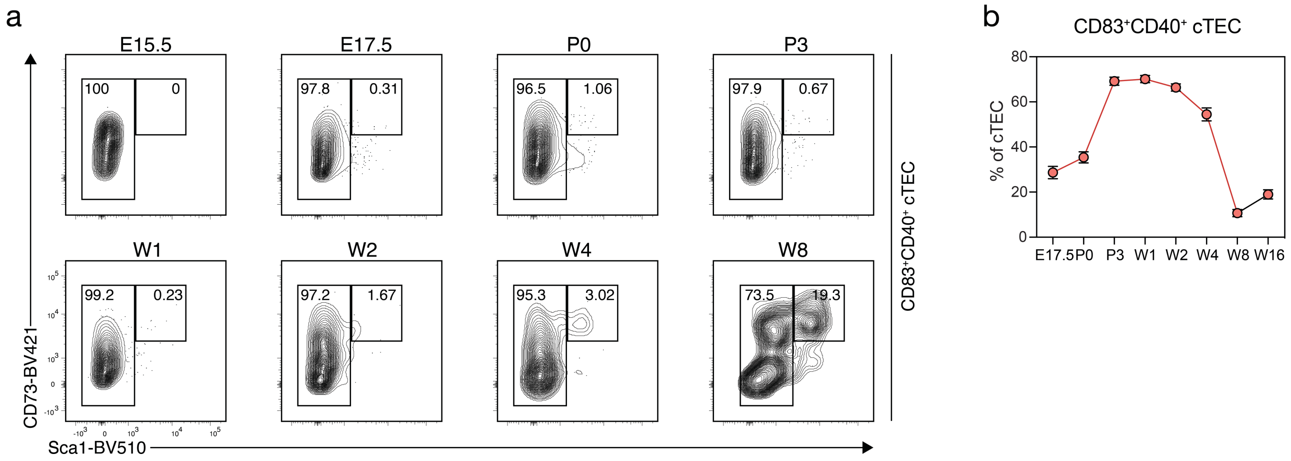

Supplementary Figure 3 | Characterization of perinatal cTEC

(a) Appearance of a CD73 and Sca1 double positive population within perinatal cTEC was analysed at the indicated timepoints in C57BL/6 WT mice. Shown are representative FACS plots. **(b)** Abundance of a CD83 and CD40 double positive population (hereafter perinatal cTEC) within cTEC was analysed at the indicated timepoints in C57BL/6 WT mice. Shown are cumulative data depicting the percent of perinatal cTEC within cTEC (E15.5 n = 13, E17.5 n = 7, P0 n = 6, P3 n = 8, W1 n = 4, W2 n = 7, W4 n = 5, W8 n = 5, W16 n = 8, from 2-3 independent experiments per timepoint). Data are presented as mean values +/– SEM. Source data are provided as a Source Data file.

Supplementary Figure 4

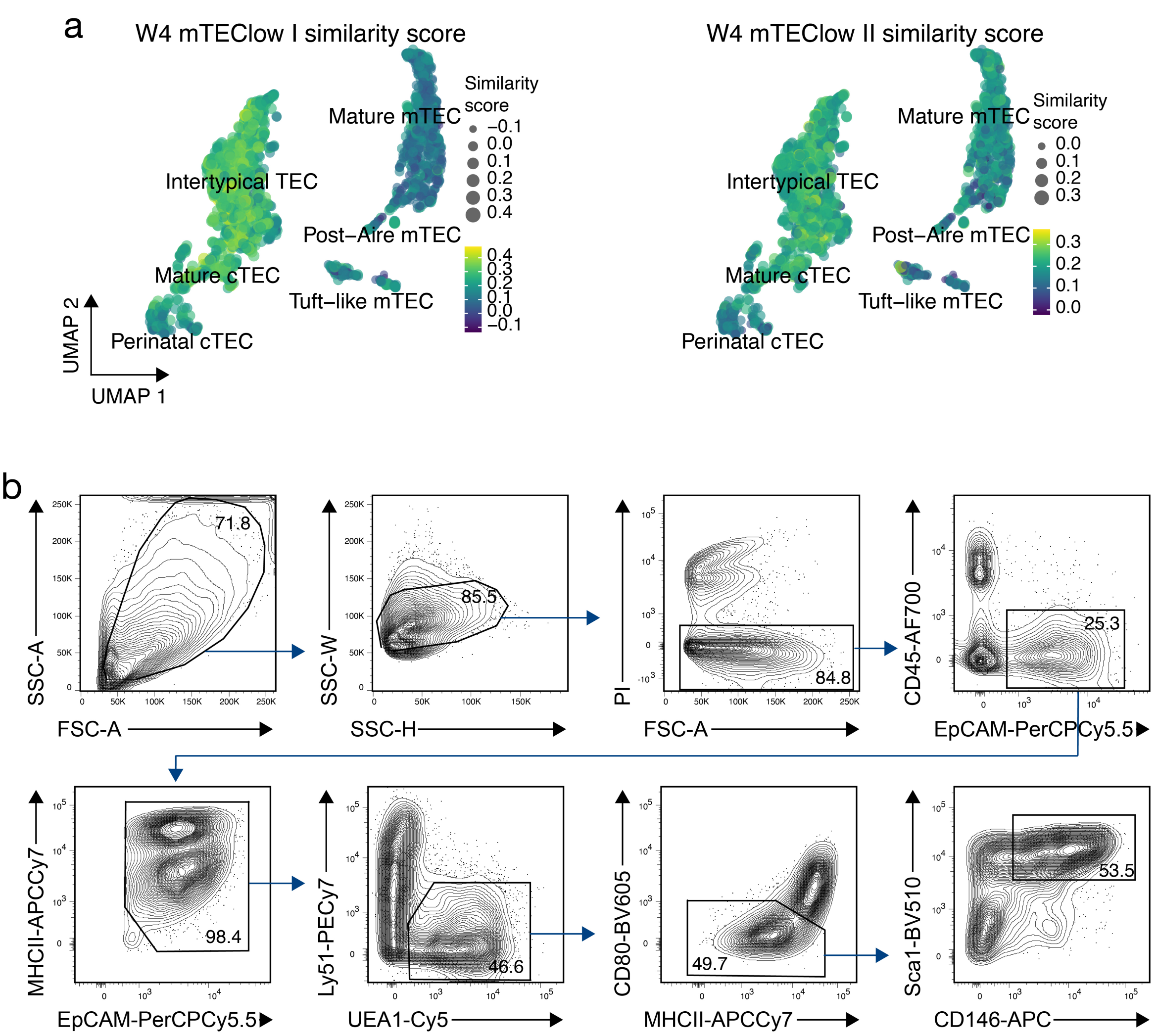

Supplementary Figure 4 | Dissecting mTEC^{lo} heterogeneity

(a) UMAP graph illustrating the similarity score of the mTEC^{lo} I and II clusters from the 4-week Infinity Flow datasets to each cell of the scRNAseq reference dataset, based on the surface protein expression levels imputed by Infinity Flow. (b) Shown are representative FACS plots illustrating the gating strategy to identify Sca1^{+}CD146^{+} cells within mTEC^{lo}. Data are derived from a 16-week-old C57BL/6 WT mouse.

Supplementary Figure 5

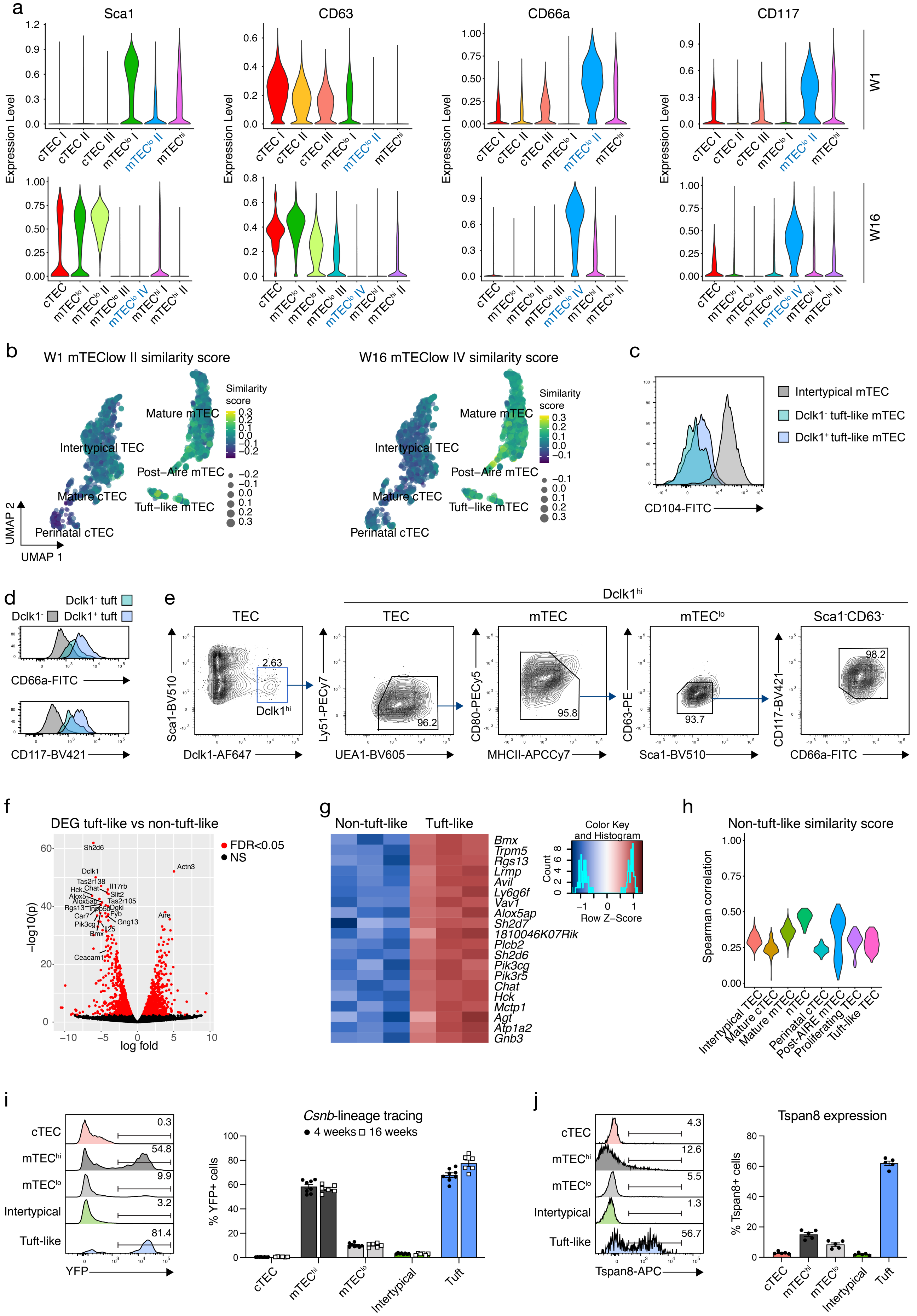

Supplementary Figure 5 | Pre-mature and post-Aire mTEC compartments

(a) Violin plots illustrating the expression of Sca1, CD63, CD66a, and CD117 on TEC from 1- and 4-week-old mice. Colours represent the different clusters, as defined in Figure 1b. **(b)** UMAP graph illustrating the similarity score of the $mTEC^{lo}$ II cluster from the 1- (left panel) and the $mTEC^{lo}$ IV cluster from the 4-week (right panel) Infinity Flow datasets to each cell of the scRNAseq reference dataset, based on the surface protein expression levels imputed by Infinity Flow. **(c)** Histgram showing the expression of CD104 in Dclk1 negative and positive tuft-like compared to intertypical mTEC. **(d)** Histograms illustrating the expression levels of CD66a and CD117 within $Dclk1^{-}$ TEC, $Dclk1^{-}$ tuft-like mTEC and $Dclk1^{+}$ tuft-like mTEC. **(e)** FACS plots illustrating the percent of $Dclk1^{+}$ TEC (first graph gated on $PI^{-}CD45^{-}EpCAM1^{+}$ cells) falling within the new tuft-like mTEC gating strategy, as defined in Figure 5d. **(f-h)** Triplicates of $Sca1^{-}CD63^{-}CD66a^{+}CD117^{+}$ tuft-like and $Sca1^{-}CD63^{-}CD66a^{-}CD117^{-}$ non-tuft-like mTEC isolated from 6-week-old WT mice were used for bulk RNAseq as described in the methods. In (f) a volcano plot depicts the differentially expressed genes between non-tuft and tuft samples. Differential gene expression analysis was conducted using the two-sided likelihood ratio test in edgeR with Benjamini-Hochberg correction for multiple hypothesis testing. In (g) a heatmap shows the expression levels of the top 20 genes associated with a tuft-like mTEC signature[1] across the samples. In (h) a violin plot depicts the Spearman correlation of the non-tuft gene signature with the previously defined TEC subpopulations from a reference scRNAseq dataset[1]. **(i)** *Csnb*^{Cre}::Rosa26$^{LSL-YFP}$ mice were analysed for the abundance of YFP^{+} cells within cTEC, $mTEC^{hi}$, $mTEC^{lo}$, intertypical TEC and tuft-like mTEC at 4 and 16 weeks after birth. Shown are representative histograms and cumulative data (4 weeks n = 8, 16 weeks n = 6, from two independent experiments per timepoint). Data are presented as mean values +/– SEM. Source data are provided as a Source Data file. **(j)** C57BL/6 WT mice were analysed for the abundance of $Tspan8^{+}$ cells within cTEC, $mTEC^{hi}$, $mTEC^{lo}$, intertypical TEC and tuft-like mTEC. Shown are representative histograms and cumulative data (n = 5, from two independent experiments). Data are presented as mean values +/– SEM. Source data are provided as a Source Data file.

Supplementary Figure 6

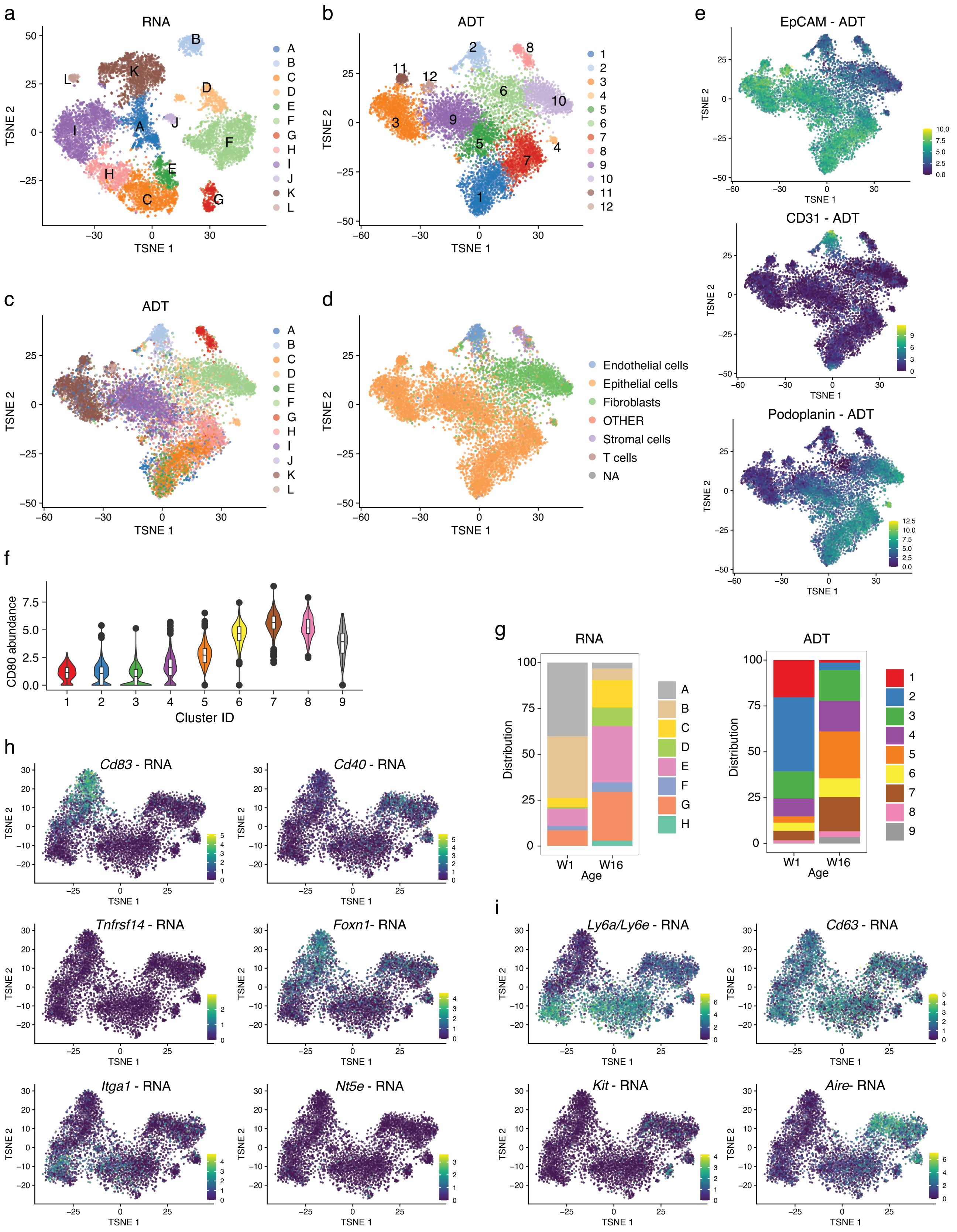

Supplementary Figure 6 | CITEseq analysis on thymic stromal cells

$CD45^{-}Ter119^{-}$ thymic stromal cells isolated from 1- and 16-week-old C57BL/6 WT mice were used for scRNAseq in combination with CITEseq as described in the methods. **(a-c)** Hierarchical clustering analysis was performed on 9953 cells either using (a) the gene expression analysis or (b) only considering the detection of ADTs. Results were projected in a 2D space using t-SNE. Each colour represents a specific cluster. In (c) t-SNE distribution of the ADT clustering is

shown using the cluster colouring of the RNA analysis. **(d)** Cells were annotated based on transcriptional similarity to reference datasets derived from the Immunological Genome Project (ImmGen). Each colour represents a specific subset as defined in the reference dataset. **(e)** T-SNE plots illustrating the scaled expression of EpCAM1, CD31, and Podoplanin across ADT clusters. **(f)** Violin plots depicting the abundance of CD80 ADTs across ADT TEC clusters. The box was drawn from the 25th percentile (Q1) to the 75th percentile (Q3) of the ADT abundance in cells from a specific cluster with the horizontal line denoting the median value. The difference Q3-Q1 forms the interquartile range (IQR). Whiskers are drawn up to the largest data point and down to the smallest data point falling within the range 1.5*IQR. All other observed data points outside the boundary of the whiskers are plotted individually as outliers. **(g)** Bar graphs illustrating the distribution of 1- and 16-week-old derived TEC across ADT (left panel) and RNA (right panel) clusters. Each colour represents a specific cluster. **(h,i)** T-SNE plots illustrating the scaled expression of the gene expression of (h) perinatal cTEC markers such as *Cd83*, *Cd40*, *Tnfrsf14*, *Foxn1*, *Itga1*, and *Nt5e*, and of (i) tuft-like and intertypical TEC markers such as *Ly6a/Ly6e*, *Cd63*, *Kit*, and *Aire* across ADT clusters.

Supplementary Figure 7

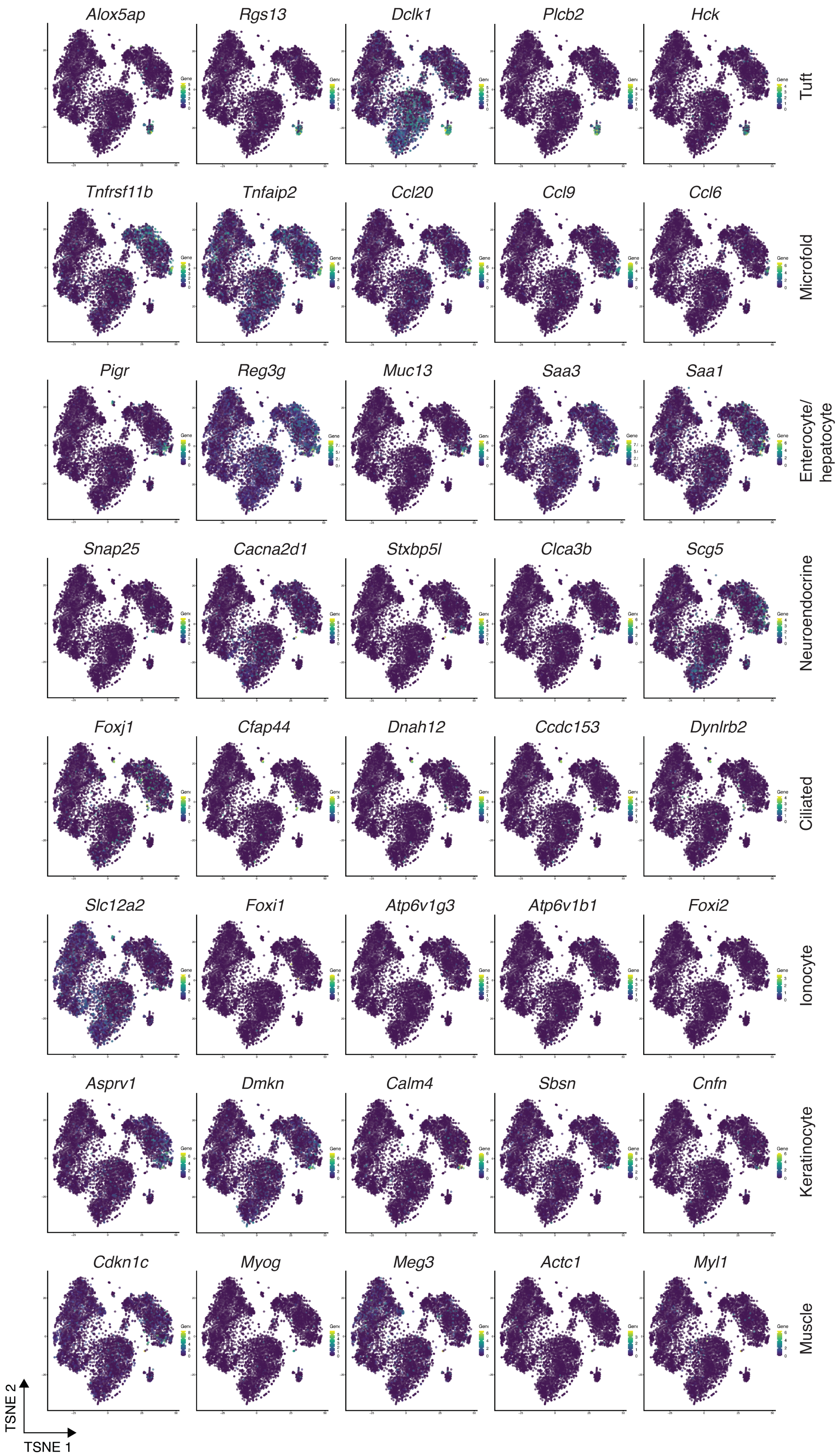

Supplementary Figure 7 | Gene signature of "mimetic" mTEC subsets

CD45^{-}Ter119^{-} thymic stromal cells isolated from 1- and 16-week-old C57BL/6 WT mice were used for scRNAseq in combination with CITEseq as described in the methods. Shown are t-SNE plots illustrating the scaled expression of the top genes described to identify the corresponding mimetic mTEC subsets[2].

Supplementary Figure 8

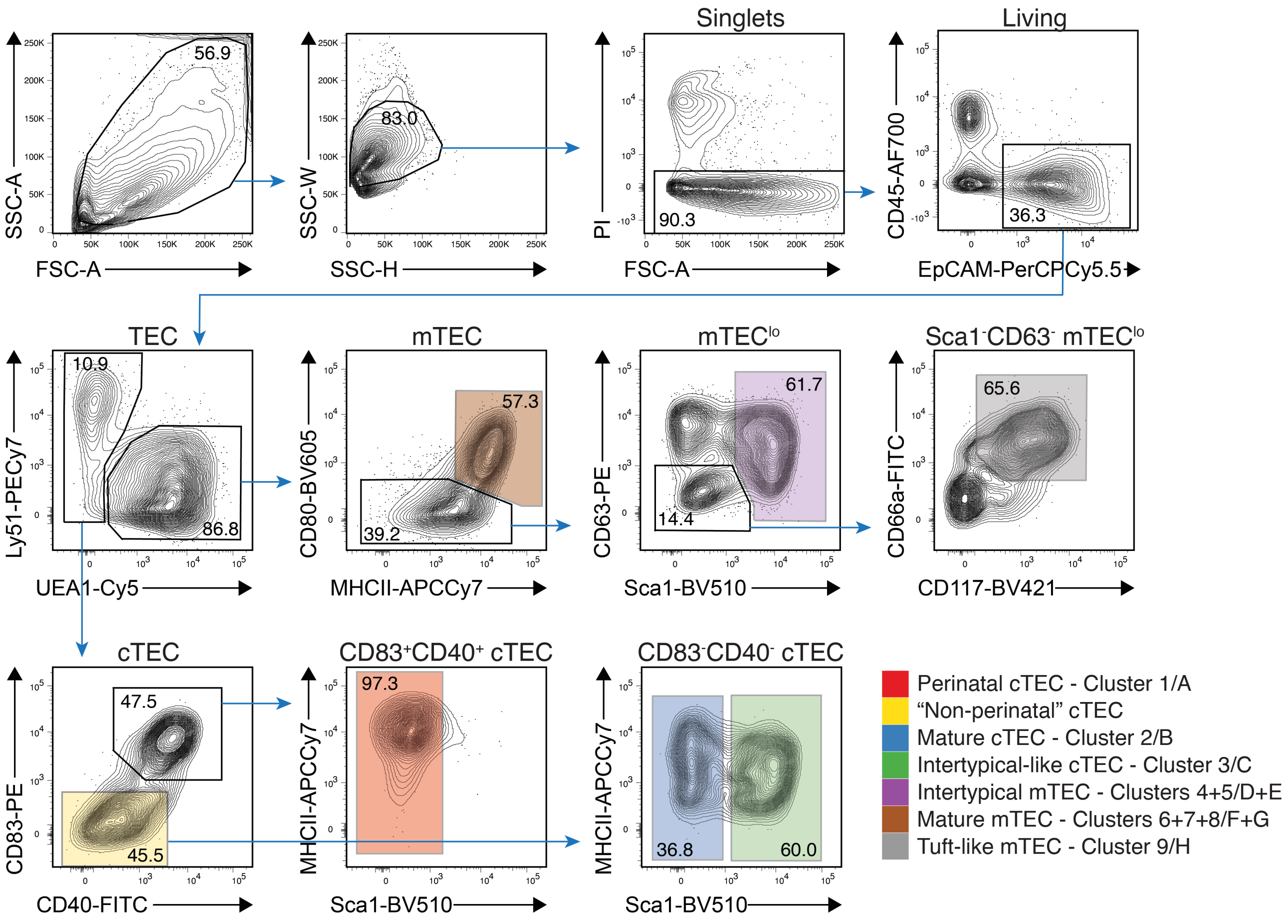

Supplementary Figure 8 | Gating strategy to identify TEC subpopulations
Shown are representative FACS plots illustrating the new gating strategy to identify TEC subpopulations based on the surface expression profiles obtained from CITEseq. Colours represent different TEC subpopulations and CITEseq clusters as indicated. Data are derived from a 4-week-old C57BL/6 WT mouse and is representative of 3 independent experiments.

Supplementary Figure 9

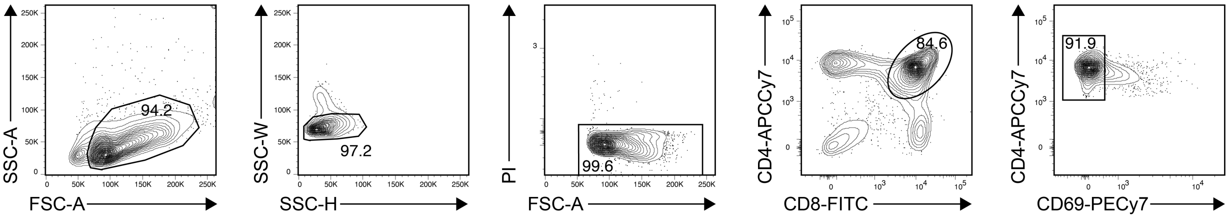

Supplementary Figure 9 | Gating strategy to identify DP thymocytes
Shown are representative FACS plots illustrating the gating strategy to identify CD69^{-} DP thymocytes. Data are derived from a 4-week-old C57BL/6 WT mouse.

Supplementary Figure 10

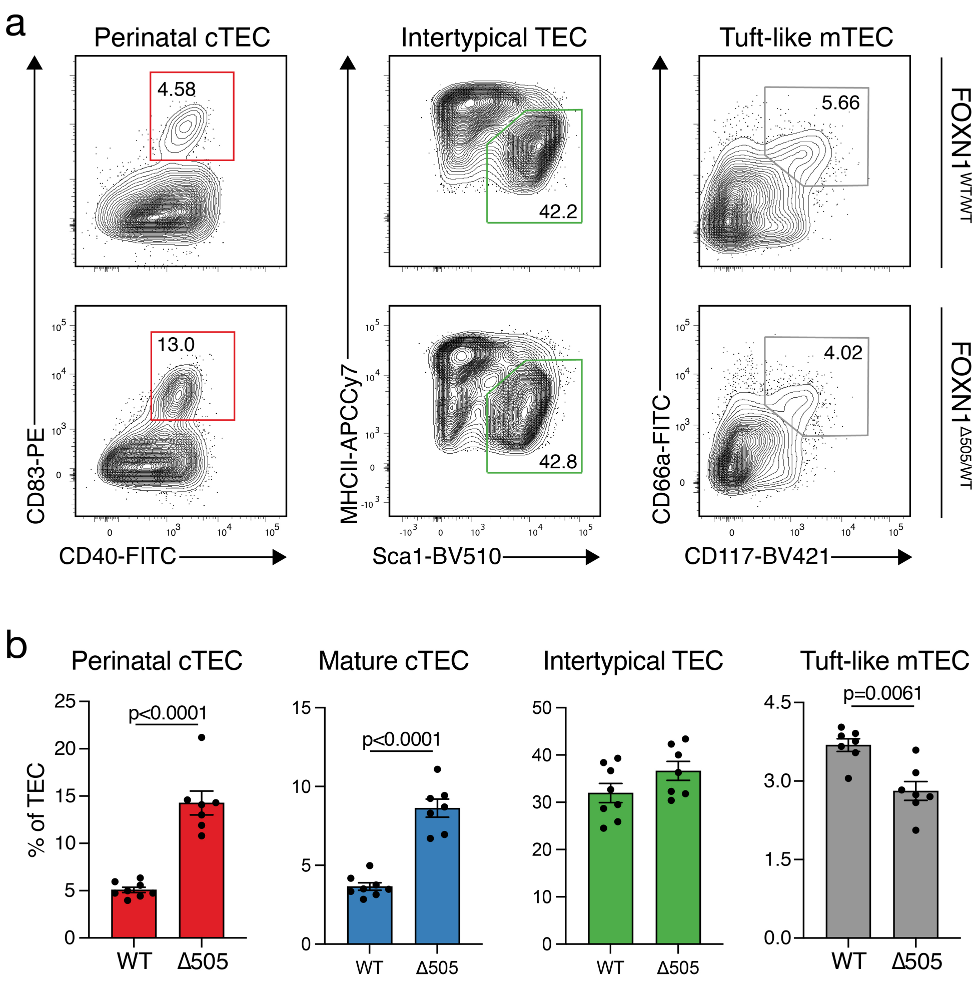

Supplementary Figure 10 | Application of new TEC markers on FOXN1$^{\Delta505/WT}$ mice

(a,b) FOXN1$^{\Delta505/WT}$ mice were analysed for the abundance of perinatal cTEC, mature cTEC, intertypical TEC and tuft-like mTEC using the new markers and compared to C57BL/6 WT mice at an age of 4-weeks. Shown are (a) representative FACS plots and (b) cumulative data (WT n = 8 (perinatal cTEC, mature cTEC, intertypicl TEC) or n = 7 (tuft-like mTEC), Δ505 n = 7, from three independent experiments). Data are presented as mean values +/– SEM. Statistical analysis was done with two-tailed unpaired Student`s t-test.

Supplementary Table 1

Exploratory markers	Isotype	Expression in thymic stromal compartment
Blank	Blank	No
AHIgG	AHIgG	No
CD3e	AHIgG	Yes
CD80	AHIgG	Yes
CD154	AHIgG	No
Notch 1	AHIgG	No
CD30	AHIgG	No
CD178	AHIgG	No
CD103	AHIgG	No
Delta-like 4	AHIgG	No
CD195	AHIgG	No
Notch 4	AHIgG	No
CD229 (Ly-9)	AHIgG	No
CD69	AHIgG	No
Notch 3	AHIgG	No
JAML	AHIgG	No
Notch 2	AHIgG	No
CD194	AHIgG	No
CD152	AHIgG	No
CD120a	AHIgG	Yes
CD11c	AHIgG	No
Delta-like 1	AHIgG	No
CD196	AHIgG	No
CD29	AHIgG	Yes
CD55	AHIgG	Yes
Jagged 2	AHIgG	No
CD79b	AHIgG	No
IFN-g R b chain	AHIgG	Yes
CD61	AHIgG	Yes
CD121a	AHIgG	Yes
TCR b chain	AHIgG	Yes
FceRIa	AHIgG	No
CD16.2	AHIgG	No
CD36	AHIgG	Yes
DcTRAIL-R1	AHIgG	Yes
CD84	AHIgG	No
CD48	AHIgG	Yes
CD49b	AHIgG	Yes
CD120b	AHIgG	No
CD183	AHIgG	No
CD262	AHIgG	Yes
HVEM	AHIgG	Yes
TCR Vg1.1 + Vg1.2	AHIgG	No
B7-H4	AHIgG	No
CD339	AHIgG	No
CD49a	AHIgG	Yes
PD-1H	AHIgG	No
CD85k	AHIgG	No
Plexin B2	AHIgG	Yes
CD27	AHIgG	No
DR3	AHIgG	No
TCR g/d	AHIgG	No
mIgG2ak	mIgG2ak	No
CD45.1	mIgG2ak	No
CD45.2	mIgG2ak	No
NK-1.1	mIgG2ak	No
Ly108	mIgG2ak	No

CD207	mIgG2ak	No
CX3CR1	mIgG2ak	Yes
mIgG1k	mIgG1k	No
CD66a	mIgG1k	Yes
IFNAR-1	mIgG1k	No
Tim-2	mIgG1k	No
CD272	mIgG1k	No
CD64	mIgG1k	Yes
CD351	mIgG1k	No
LAP	mIgG1k	No
TIGIT	mIgG1k	No
Trem-like 4	mIgG1k	No
CD59a	mIgG1k	Yes
Ly49H	mIgG1k	No
CD90.1	mIgG1k	No
mIgG2bk	mIgG2bk	No
CD157	mIgG2bk	Yes
CD159a	mIgG2bk	Yes
XCR1	mIgG2bk	No
mIgMk	mIgMk	No
SSEA-1	mIgMk	Yes
rIgG1k	rIgG1k	No
Ig light chain _	rIgG1k	No
Siglec H	rIgG1k	No
CD255	rIgG1k	No
CD202b	rIgG1k	Yes
GITR Ligand	rIgG1k	No
CD147	rIgG1k	Yes
CD73	rIgG1k	Yes
CD51	rIgG1k	Yes
NKG2D	rIgG1k	No
CD96	rIgG1k	No
Integrin b7	rIgG1k	No
CD210	rIgG1k	No
CD83	rIgG1k	Yes
Mac-3	rIgG1k	Yes
CD223	rIgG1k	No
CD134	rIgG1k	No
Blank	Blank	No
CD41	rIgG1k	No
CD268	rIgG1k	No
CD144	rIgG1k	No
CD370	rIgG1k	No
CD369 (Dectin-1,CLEC7A)	rIgG1k	No
PIR-A/B	rIgG1k	No
CD22	rIgG1k	No
E-Cadherin	rIgG1k	Yes
CD172a (SIRPa)	rIgG1k	Yes
CD319	rIgG1k	Yes
rIgG2a	rIgG2a	No
MAIR-V	rIgG2a	No
CD146	rIgG2a	Yes
VISTA	rIgG2a	Yes
CD8a	rIgG2a	Yes
CD275	rIgG2a	No
CD34	rIgG2a	Yes
Ly-6A/E	rIgG2a	Yes
CD40	rIgG2a	Yes
CD45R/B220	rIgG2a	No
CD197	rIgG2a	No

CD47	rIgG2a	Yes
CD98	rIgG2a	Yes
CD14	rIgG2a	Yes
CD107a (LAMP-1)	rIgG2a	Yes
CD18	rIgG2a	Yes
Ly-6G	rIgG2a	Yes
CD21/35	rIgG2a	No
Mac-2	rIgG2a	No
CD199	rIgG2a	No
Ly-51	rIgG2a	Yes
IgD	rIgG2a	No
Tim-4	rIgG2a	No
CD71	rIgG2a	Yes
H-2	rIgG2a	Yes
CD45RB	rIgG2a	Yes
CD326	rIgG2a	Yes
IgM	rIgG2a	No
CD155	rIgG2a	Yes
CD200R	rIgG2a	No
CD254	rIgG2a	No
IL-21R	rIgG2a	No
CD276	rIgG2a	No
CD9	rIgG2a	Yes
CD105	rIgG2a	Yes
CD366	rIgG2a	No
4-1BB Ligand	rIgG2a	No
CD265	rIgG2a	No
TLR4 (CD284)/MD2 Complex	rIgG2a	Yes
CD19	rIgG2a	No
LPAM-1	rIgG2a	No
CD62L	rIgG2a	No
CD23	rIgG2a	No
CD5	rIgG2a	Yes
CD273	rIgG2a	Yes
CD31	rIgG2a	Yes
F4/80	rIgG2a	No
CD94	rIgG2a	No
CD267	rIgG2a	No
Ly-49A	rIgG2a	No
CD180	rIgG2a	Yes
CD11a	rIgG2a	Yes
LT beta R	rIgG2a	Yes
CD122	rIgG2a	No
CD106	rIgG2a	Yes
CD365	rIgG2a	No
CD115	rIgG2a	No
CD140a	rIgG2a	Yes
PDC-TREM	rIgG2a	No
CD135	rIgG2a	No
CD127	rIgG2a	No
CD140b	rIgG2a	Yes
ESAM	rIgG2a	Yes
CD200	rIgG2a	Yes
CD309	rIgG2a	No
TLT-2	rIgG2a	No
CD253	rIgG2a	No
CD335	rIgG2a	No
CD205	rIgG2a	Yes
Galectin-9	rIgG2a	Yes
CD200R3	rIgG2a	No

MAIR-IV	rIgG2a	No
Ly49D	rIgG2a	No
CD123	rIgG2a	No
CD355	rIgG2a	No
CD169	rIgG2a	No
CD138	rIgG2a	No
CD160	rIgG2a	No
CD39	rIgG2a	Yes
GARP	rIgG2a	No
CD179a	rIgG2a	No
CD371	rIgG2a	No
CD63	rIgG2a	Yes
CD49e	rIgG2a	No
CD193	rIgG2a	No
RbIgG	RbIgG	No
CD300LG	rIgG2a	Yes
CD301a	rIgG2a	No
IL-33Ra	rIgG2a	No
CD304	rIgG2a	No
CD6	rIgG2a	No
CD100	rIgG2a	No
CD104	rIgG2a	Yes
CD182	rIgG2a	Yes
MAdCAM-1	rIgG2a	Yes
MERTK (Mer)	rIgG2a	Yes
CD226	rIgG2a	No
Ly6K	rIgG2a	Yes
CD16/32	rIgG2a	Yes
CD150	rIgG2a	Yes
CD25	rIgG2a	Yes
CD38	rIgG2a	Yes
CD133	rIgG2a	Yes
CD301b	rIgG2a	No
CD34	rIgG2a	Yes
rIgG2bk	rIgG2bk	No
CD43	rIgG2bk	Yes
FR4	rIgG2bk	No
CD1d	rIgG2bk	Yes
CD70	rIgG2bk	Yes
CD4	rIgG2bk	Yes
I-A/I-E	rIgG2bk	Yes
CD153	rIgG2bk	No
CD54	rIgG2bk	Yes
33D1	rIgG2bk	No
CD90.2	rIgG2bk	Yes
TER-119	rIgG2bk	No
CD49d	rIgG2bk	Yes
CD24	rIgG2bk	Yes
Ly-6G/Ly-6C	rIgG2bk	Yes
CD86	rIgG2bk	Yes
CD11b	rIgG2bk	Yes
CD45	rIgG2bk	Yes
CD279	rIgG2bk	No
RAE-1g	rIgG2bk	No
CD8b	rIgG2bk	Yes
CD44	rIgG2bk	Yes
CD126	rIgG2bk	No
CD317	rIgG2bk	Yes
CD132	rIgG2bk	Yes
CD3	rIgG2bk	Yes

CD274	rIgG2bk	Yes
CD117	rIgG2bk	Yes
CD93	rIgG2bk	No
CD252	rIgG2bk	No
MD-1	rIgG2bk	No
CD357	rIgG2bk	Yes
CD185	rIgG2bk	No
CD300c/d	rIgG2bk	No
CD186 (CXCR6)	rIgG2bk	No
CD130	rIgG2bk	Yes
CD198	rIgG2bk	No
CD20	rIgG2bk	No
CD124	rIgG2bk	Yes
IL-23R	rIgG2bk	No
CD184	rIgG2bk	Yes
CD2	rIgG2bk	No
rIgG2ck	rIgG2ck	No
Ly-6C	rIgG2ck	Yes
Ly-6D	rIgG2ck	Yes
rIgMk	rIgMk	No
CD49b	rIgMk	Yes
GL7	rIgMk	Yes
SHIgG	SHIgG	No
CD28	SHIgG	No
Podoplanin	SHIgG	Yes
CD137	SHIgG	No
CD278	SHIgG	No
KLRG1	SHIgG	No
Ly-49C/F/I/H	SHIgG	No
CD177	RbIgG	No
F3	RbIgG	Yes
Gp2	rIgG2a	Yes
Tspan8	rIgG2bk	Yes
Foxn1	mIgG2bk	Yes

Supplementary Table 1 | Infinity Flow exploratory markers, the isotype of the corresponding antibodies and the expression status on thymic stromal cells.

Supplementary Table 2

LEGENDScreen protein	Gene
CD3e.XGBoost-bgc	*Cd3e*
CD80.XGBoost-bgc	*Cd80*
CD81.XGBoost-bgc	*Cd81*
CD120a.XGBoost-bgc	*Tnfrsf1a*
CD29.XGBoost-bgc	*Itgb1*
CD55.XGBoost-bgc	*Cd55*
IFN-g R b chain.XGBoost-bgc	*Ifngr2*
CD61.XGBoost-bgc	*Itgb3*
CD121a.XGBoost-bgc	*Il1r1*
TCR b chain.XGBoost-bgc	*Tcrb*
CD36.XGBoost-bgc	*Cd36*
DcTRAIL-R1.XGBoost-bgc	*Tnfrsf23*
CD48.XGBoost-bgc	*Cd48*
CD49b.XGBoost-bgc	*Itga2*
CD262.XGBoost-bgc	*Tnfrsf10b*
HVEM.XGBoost-bgc	*Tnfrsf14*
CD49a.XGBoost-bgc	*Itga1*
Plexin B2.XGBoost-bgc	*Plxnb2*
CX3CR1.XGBoost-bgc	*Cx3cr1*
CD66a.XGBoost-bgc	*Ceacam1*
CD64.XGBoost-bgc	*Fcgr1a*
CD59a.XGBoost-bgc	*Cd59a*
CD157.XGBoost-bgc	*Bst1*
CD159a.XGBoost-bgc	*Klrc1*
SSEA-1.XGBoost-bgc	*Fut4*
CD202b.XGBoost-bgc	*Tek*
CD147.XGBoost-bgc	*Bsg*
CD73.XGBoost-bgc	*Nt5e*
CD51.XGBoost-bgc	*Itgav*
CD83.XGBoost-bgc	*Cd83*
Mac-3.XGBoost-bgc	*Lgals3*
E-Cadherin.XGBoost-bgc	*Cdh1*
CD172a (SIRPa).XGBoost-bgc	*Sirpa*
CD319.XGBoost-bgc	*Slamf7*
CD146.XGBoost-bgc	*Mcam*
VISTA.XGBoost-bgc	*Vsir*
CD8a.XGBoost-bgc	*Cd8a*
CD34.XGBoost-bgc	*Cd34*
Ly-6A/E.XGBoost-bgc	*Ly6a*
CD40.XGBoost-bgc	*Cd40*
CD47.XGBoost-bgc	*Cd47*
CD98.XGBoost-bgc	*Slc3a2*
CD14.XGBoost-bgc	*Cd14*
CD107a (LAMP-1).XGBoost-bgc	*Lamp1*
CD18.XGBoost-bgc	*Itgb2*
Ly-6G.XGBoost-bgc	*Ly6g*
Ly-51.XGBoost-bgc	*Enpep*
CD71.XGBoost-bgc	*Tfrc*
H-2.XGBoost-bgc	
CD45RB.XGBoost-bgc	*Ptprc*
CD326.XGBoost-bgc	*Epcam*
CD155.XGBoost-bgc	*Pvr*
CD9.XGBoost-bgc	*Cd9*
CD105.XGBoost-bgc	*Eng*

TLR4 (CD284)/MD2 Complex.XGBoost-bgc	*Tlr4*
CD5.XGBoost-bgc	*Cd5*
CD273.XGBoost-bgc	*Pdcd1lg2*
CD31.XGBoost-bgc	*Pecam1*
CD180.XGBoost-bgc	*Cd180*
CD11a.XGBoost-bgc	*Itgal*
LT beta R.XGBoost-bgc	*Ltbr*
CD106.XGBoost-bgc	*Vcam1*
CD140a.XGBoost-bgc	*Pdgfra*
CD140b.XGBoost-bgc	*Pdgfrb*
ESAM.XGBoost-bgc	*Esam*
CD200.XGBoost-bgc	*Cd200*
CD205.XGBoost-bgc	*Ly75*
Galectin-9.XGBoost-bgc	*Lgals9*
CD39.XGBoost-bgc	*Entpd1*
CD63.XGBoost-bgc	*Cd63*
CD300LG.XGBoost-bgc	*Cd300lg*
CD104.XGBoost-bgc	*Itgb4*
CD182.XGBoost-bgc	*Cxcr2*
MAdCAM-1.XGBoost-bgc	*Madcam1*
MERTK (Mer).XGBoost-bgc	*Merk*
Ly6K.XGBoost-bgc	*Ly6k*
CD16/32.XGBoost-bgc	*Fcgr3a*
CD150.XGBoost-bgc	*Slamf1*
CD25.XGBoost-bgc	*Il2ra*
CD38.XGBoost-bgc	*Cd38*
CD133.XGBoost-bgc	*Prom1*
CD43.XGBoost-bgc	*Spn*
CD1d.XGBoost-bgc	*Cd1d*
CD70.XGBoost-bgc	*Cd70*
CD4.XGBoost-bgc	*Cd4*
I-A/I-E.XGBoost-bgc	*H2-Ab1*
CD54.XGBoost-bgc	*Icam1*
CD90.2.XGBoost-bgc	*Thy1*
CD49d.XGBoost-bgc	*Itga4*
CD24.XGBoost-bgc	*Cd24a*
Ly-6G/Ly-6C.XGBoost-bgc	*Ly6g*
CD86.XGBoost-bgc	*Cd86*
CD11b.XGBoost-bgc	*Itgam*
CD45.XGBoost-bgc	*Ptprc*
CD8b.XGBoost-bgc	*Cd8b*
CD44.XGBoost-bgc	*Cd44*
CD317.XGBoost-bgc	*Bst2*
CD132.XGBoost-bgc	*Il2rg*
CD3.XGBoost-bgc	*Cd3g*
CD274.XGBoost-bgc	*Cd274*
CD117.XGBoost-bgc	*Kit*
CD357.XGBoost-bgc	*Tnfrsf18*
CD130.XGBoost-bgc	*Il6st*
CD124.XGBoost-bgc	*Il4ra*
CD184.XGBoost-bgc	*Cxcr4*
Ly-6C.XGBoost-bgc	*Ly6c1*
Ly-6D.XGBoost-bgc	*Ly6d*
GL7.XGBoost-bgc	*Ly77*
Podoplanin.XGBoost-bgc	*Pdpn*
F3.XGBoost-bgc	*F3*
Gp2.XGBoost-bgc	*Gp2*

Tspan8.XGBoost-bgc	*Tspan8*
Foxn1.XGBoost-bgc	*Foxn1*
Podoplanin	*Pdpn*
Aire	*Aire*
UEA1	*Fut1*
MHCII	*H2-Ab1*
CD80	*Cd80*
CD86	*Cd86*
Sca1	*Ly6a*
CD40	*Cd40*
Ly51	*Enpep*

Supplementary Table 2 | LEGENDScreen antibody epitopes and their corresponding genes used for computational comparisons.

References

1. Baran-Gale, J. *et al.* Ageing compromises mouse thymus function and remodels epithelial cell differentiation. *Elife* **9** (2020).

2. Michelson, D.A., Hase, K., Kaisho, T., Benoist, C. & Mathis, D. Thymic epithelial cells co-opt lineage-defining transcription factors to eliminate autoreactive T cells. *Cell* **185**, 2542-2558 e2518 (2022).
