# Supplementary material for: Combined multidimensional single-cell protein and RNA profiling dissects the cellular and functional heterogeneity of thymic epithelial cells
Source: Nat Commun. 2023 Jul 10;14:4071. doi: 10.1038/s41467-023-39722-9 (PMC10333192; doi:10.1038/s41467-023-39722-9)
Supplement: Supplementary file 3 — Reporting Summary [file 41467_2023_39722_MOESM3_ESM.pdf]

nature portfolio

Corresponding author(s): Georg A. Holländer

Last updated by author(s): 2023 - 06 - 06

Reporting Summary

Nature Portfolio wishes to improve the reproducibility of the work that we publish. This form provides structure for consistency and transparency in reporting. For further information on Nature Portfolio policies, see our Editorial Policies and the Editorial Policy Checklist.

Statistics

For all statistical analyses, confirm that the following items are present in the figure legend, table legend, main text, or Methods section.

n/a	Confirmed	
☐	☒	The exact sample size (*n*) for each experimental group/condition, given as a discrete number and unit of measurement
☒	☐	A statement on whether measurements were taken from distinct samples or whether the same sample was measured repeatedly
☐	☒	The statistical test(s) used AND whether they are one- or two-sided<br>*Only common tests should be described solely by name; describe more complex techniques in the Methods section.*
☒	☐	A description of all covariates tested
☒	☐	A description of any assumptions or corrections, such as tests of normality and adjustment for multiple comparisons
☐	☒	A full description of the statistical parameters including central tendency (e.g. means) or other basic estimates (e.g. regression coefficient) AND variation (e.g. standard deviation) or associated estimates of uncertainty (e.g. confidence intervals)
☐	☒	For null hypothesis testing, the test statistic (e.g. *F*, *t*, *r*) with confidence intervals, effect sizes, degrees of freedom and *P* value noted<br>*Give P values as exact values whenever suitable.*
☒	☐	For Bayesian analysis, information on the choice of priors and Markov chain Monte Carlo settings
☐	☒	For hierarchical and complex designs, identification of the appropriate level for tests and full reporting of outcomes
☐	☒	Estimates of effect sizes (e.g. Cohen's *d*, Pearson's *r*), indicating how they were calculated

Our web collection on statistics for biologists contains articles on many of the points above.

Software and code

Policy information about availability of computer code

Data collection	Flow cytometry and cell sorting were performed using FACS Diva using BD LSRFortessa and BD FACSAria III.<br>Images were acquired using a Leica DMi8 microscope.<br>For CITE-seq single-cells were captured using a single 10X Genomics Chromium Single Cell Controller and libraries were sequenced on an Illumina NovaSeq 6000 instrument.
Data analysis	Flow cytometry data was analyzed on FlowJo v10.<br>Graphs and statistics were created and analyzed using GraphPad Prism Version 9 with the exception of the Infinity Flow, bulk RNAseq and CITE-seq datasets. Infinity Flow and scRNAseq were analyzed using R (v4.1.2) by applying the Seurat package.<br>CITE-seq cDNA reads were aligned to 'mm10' genome using Ensembl 102 gene models with the STARsolo tool (v2.7.9a). ADT libraries were also processed using the STARsolo tool. Further analysis steps were performed using R (v4.1.2). Multiple Bioconductor (v3.14) packages including DropletUtils (v1.14.2), scDblFinder (v1.8.0), scran (v1.22.1), scater (v1.22.0), scuttle (1.4.0) and batchelor (v1.10.0) were applied for the further analysis of the dataset. Normalised 39 log-count values for the gene expression were used to construct a shared nearest-neighbour graph, which nodes, i.e. cells, were clustered by 'cluster_louvain' method from the R igraph package. The data set was subjected to the cell-type annotation using the Bioconductor package SingleR (v1.8.1) and samples from the Immunological Genome Project (ImmGen) provided by the Bioconducter package celldex (v1.4.0) as the reference.<br>For bulk RNAseq analysis reads were trimmed using Trimmomatic (version 0.36) to remove adapter sequences and aligned to the mouse genome (mm10) using STAR (version 2.7.3a). HTSeq (version 0.12.4) was used to assign reads to genes with the option "intersection-nonempty". Differentially expressed genes were identified using edgeR (version 3.40.2) (FDR < 0.05).

For manuscripts utilizing custom algorithms or software that are central to the research but not yet described in published literature, software must be made available to editors and reviewers. We strongly encourage code deposition in a community repository (e.g. GitHub). See the Nature Portfolio guidelines for submitting code & software for further information.

Data

Policy information about availability of data

All manuscripts must include a data availability statement. This statement should provide the following information, where applicable:

- Accession codes, unique identifiers, or web links for publicly available datasets
- A description of any restrictions on data availability
- For clinical datasets or third party data, please ensure that the statement adheres to our policy

The CITEseq and the bulk RNAseq datasets have been deposited in the Gene Expression Omnibus database under accession numbers “GSE215418 [https://www.ncbi.nlm.nih.gov/geo/query/acc.cgi?acc=GSE215418] and “GSE226128 [https://www.ncbi.nlm.nih.gov/geo/query/acc.cgi?acc=GSE226128]”, respectively.

Human research participants

Policy information about studies involving human research participants and Sex and Gender in Research.

Reporting on sex and gender	n/a
Population characteristics	n/a
Recruitment	n/a
Ethics oversight	n/a

Note that full information on the approval of the study protocol must also be provided in the manuscript.

Field-specific reporting

Please select the one below that is the best fit for your research. If you are not sure, read the appropriate sections before making your selection.

[x] Life sciences [ ] Behavioural & social sciences [ ] Ecological, evolutionary & environmental sciences

For a reference copy of the document with all sections, see nature.com/documents/nr-reporting-summary-flat.pdf

Life sciences study design

All studies must disclose on these points even when the disclosure is negative.

Sample size	Sample size was chosen based on pilot experiments and previous experience with similar experiments to achieve sufficient statistical power. Data were collected from two or more biological replicates.
Data exclusions	No data was excluded from analysis.
Replication	All experiments were repeated and reliably reproduced. Data were collected from three biological replicates if not stated differently. Number of independent experiments performed are indicated in the figure legends.
Randomization	No randomization of mice was performed. Litter mates, where applicable, or age- and gender-matched mice were used as controls.
Blinding	Blinding was not undertaken as only wild-type animals at distinct ages were analyzed with the exception of data shown in Figure 4f,g and Supplementary Figure 10 were gene targeted animals were specifically and knowingly used to verify findings. The methodological approaches used, massively parallel flow cytometry and machine learning , did not explicitly allow for blinding.

Reporting for specific materials, systems and methods

We require information from authors about some types of materials, experimental systems and methods used in many studies. Here, indicate whether each material, system or method listed is relevant to your study. If you are not sure if a list item applies to your research, read the appropriate section before selecting a response.

Materials & experimental systems

n/a	Involved in the study
☐	☒ Antibodies
☒	☐ Eukaryotic cell lines
☒	☐ Palaeontology and archaeology
☐	☒ Animals and other organisms
☒	☐ Clinical data
☒	☐ Dual use research of concern

Methods

n/a	Involved in the study
☒	☐ ChIP-seq
☐	☒ Flow cytometry
☒	☐ MRI-based neuroimaging

Antibodies

Antibodies used

Target, fluorochrome/tag, clone, species reactivity, supplier, catalog number:
anti-CD3ε, unlabeled, clone KT3, Mouse, Produced in house
anti-CD4, APCCy7, clone GK1.5, Mouse, Biolegend, 100414
anti-CD4, FITC, clone GK1.5, Mouse, Biolegend, 100406
anti-CD5, PerCPCy5.5, clone 53-7.3, Mouse, Biolegend, 100624
anti-CD8α, AF700, clone 53-6.7, Mouse, Biolegend, 100730
anti-CD31, FITC, clone 390, Mouse, Biolegend, 102406
anti-CD40, FITC, clone 3/23, Mouse, Biolegend, 124608
anti-CD40, PECy5, clone 3/23, Mouse, Biolegend, 124617
anti-CD45, AF700, clone 30-F11, Mouse, Biolegend, 103128
anti-CD63, PE, clone NVG-2, Mouse, Biolegend, 143904
anti-CD66a, APC, clone MAb-CC1, Mouse, Biolegend, 134509
anti-CD66a, FITC, clone MAb-CC1, Mouse, Biolegend, 134518
anti-CD69, unlabeled ,clone H1.2F3, Mouse, Biolegend, 104502
anti-CD69, PECy5, clone H1.2F3, Mouse, Biolegend, 104510
anti-CD73, BV421, clone TY/11.8, Mouse, Biolegend, 127217
anti-CD80, BV605, clone 16-10A1, Mouse, Biolegend, 104729
anti-CD80, PECy5, clone 16-10A1, Mouse, Biolegend, 104712
anti-CD83, Bio, clone Michel-19, Mouse, Biolegend, 121503
anti-CD83, PE, clone Michel-19, Mouse, Biolegend, 121508
anti-CD86, BV650, clone GL-1, Mouse, Biolegend, 105036
anti-CD104, FITC, clone 346-11A, Biolegend, 123606
anti-CD117, BV421, clone 2B8, Mouse, BD Biosciences, 562609
anti-CD146, APC, clone ME-9F1, Mouse, Biolegend, 134712
anti-AIRE, AF750, clone 5H12, Mouse, Invitrogen, 14-5934-82
anti-EpCAM1, PerCPCy5.5, clone G8.8, Mouse, Biolegend, 118220
anti-Dclk1, unlabeled, Mouse, Abcam, ab31704
anti-HVEM, APC, clone HMHV-1B18, Mouse, Biolegend, 136305
anti-K8, unlabeled, clone TROMA-1, Mouse, NICHD, supported Hybridoma Bank
anti-K14, unlabeled, clone Poly19053, Mouse, Biolegend, 905304
anti-Ly51, unlabeled, clone 6C3, Mouse, Biolegend, 108302
anti-Ly51 ,PECy7, clone 6C3, Mouse, Biolegend, 108314
anti-MHCII, APC/Fire 750, clone M5, Mouse, Biolegend, 107652
anti-MHCII, BV510, clone M5, Mouse, Biolegend, 107636
anti-Podoplanin, APC, clone 8.1.1, Mouse, Biolegend, 127410
UEA1, Bio, Mouse, Vector Laboratories, B-1065-2
UEA1, Cy5, Mouse, Vector Laboratories, L-1060-5
anti-Sca1, BV510, clone D7, Mouse, Biolegend, 108129
anti-Sca1, BV785, clone D7, Mouse, Biolegend, 108139
anti-Ter119, FITC ,clone TER119, Mouse, Biolegend, 116206
anti-TCRβ, FITC, clone H57-597, Mouse, Biolegend, 109205
anti-TCRβ, PE, clone H57-597, Mouse, Biolegend, 109208
anti-Tspan8, APC, Mouse, R&D Systems, FAB6524A
anti-CD9, TotalSeq, clone MZ3, Mouse, Biolegend, 124819
anti-CD31, TotalSeq, clone 390, Mouse, Biolegend, 102437
anti-CD36, TotalSeq, clone HM36, Mouse, Biolegend, 102621
anti-CD40, TotalSeq, clone 3/23, Mouse, Biolegend, 124633
anti-CD49a, TotalSeq, clone HMα1, Mouse, Biolegend, 142613
anti-CD54, TotalSeq, clone YN1/1.7.4, Mouse, Biolegend, 116127
anti-CD63, TotalSeq, clone NVG-2, Mouse, Biolegend, 143915
anti-CD73, TotalSeq, clone TY/11.8, Mouse, Biolegend, 127227
anti-CD80, TotalSeq, clone 16-10A1, Mouse, Biolegend, 104745
anti-CD83, TotalSeq, clone Michel-19, Mouse, Biolegend, 121519
anti-CD86, TotalSeq, clone GL-1, Mouse, Biolegend, 105047

anti-CD117, TotalSeq, clone 2B8, Mouse, Biolegend, 105843
anti-CD133, TotalSeq, clone 15-2C11, Mouse, Biolegend, 141217
anti-CD146, TotalSeq, clone P1H12, Human, Biolegend, 361017
anti-CD157, TotalSeq, clone BP-3, Mouse, Biolegend, 140209
anti-CD200, TotalSeq, clone OX-90, Mouse, Biolegend, 123811
anti-CD274, TotalSeq, clone MIH6, Mouse, Biolegend, 153604
anti-CD300LG, TotalSeq, clone ZAQ5, Mouse, Biolegend, 147105
anti-EpCAM1, TotalSeq, clone G8.8, Mouse, Biolegend, 118237
anti-HVEM, TotalSeq, clone HMHV-1B18, Mouse, Biolegend, 136307
anti-Ly6D, TotalSeq, clone 49-H4, Mouse, Biolegend, 138603
anti-Ly6C/Ly6G, TotalSeq, clone RB6-8C5, Mouse, Biolegend, 108459
anti-MadCAM1, TotalSeq, clone MECA-367, Mouse, Biolegend, 120713
anti-MHCII, TotalSeq, clone M5, Mouse, Biolegend, 107653
anti-Podoplanin, TotalSeq, clone 8.1.1, Mouse, Biolegend, 127427
anti-Sca1, TotalSeq, clone D7, Mouse, Biolegend, 108147

Validation	Antibodies were validated and quality tested for flow cytometry as stated by the manufacturers. If required the antibody concentrations used were further optimized by titration prior to use.

Animals and other research organisms

Policy information about studies involving animals; ARRIVE guidelines recommended for reporting animal research, and Sex and Gender in Research

Laboratory animals	Species: Mus Musculus. Strains: WT C57BL/6 (analysed from E15.5 to week 16 postnatally), Csnb-Cre, Rosa26-LSL-YFP, FOXN1-D505/WT (analysed 4-weeks after birth), Foxn1-GFP (analysed 2 weeks after birth), Rag2-/- (analysed 4- and 16-weeks after birth), Pou2f3-/- (analysed 4-6 weeks after birth). Csnb-Cre mice were crossed to Rosa26-LSL-YFP (analysed at 4- and 16-weeks after birth).
Wild animals	The study did not involve wild animals.
Reporting on sex	Both male and female animals were used. Gender-matched wild-type mice were used in all experiments as a reference for genetically modified animals.
Field-collected samples	The study did not involve samples collected from the field.
Ethics oversight	Animals were maintained under specific pathogen–free conditions at the University of Oxford Biomedical Science facilities. Experiments were performed according to institutional and U.K. Home Office regulations and age- and gender-matched wild-type mice were used in all experiments as a reference for genetically modified animals.<br>Rag2-/- mice were bred and maintained in the mouse facility of the Department of Biomedicine at the University of Basel in accordance with permissions and regulations of the Cantonal Veterinary Office of Basel-Stadt.

Note that full information on the approval of the study protocol must also be provided in the manuscript.

Flow Cytometry

Plots

Confirm that:

- [x] The axis labels state the marker and fluorochrome used (e.g. CD4-FITC).
- [x] The axis scales are clearly visible. Include numbers along axes only for bottom left plot of group (a 'group' is an analysis of identical markers).
- [x] All plots are contour plots with outliers or pseudocolor plots.
- [x] A numerical value for number of cells or percentage (with statistics) is provided.

Methodology

Sample preparation	Isolated thymi were cleaned from adipose tissue, separated into the two lobes, and subsequently subjected to three rounds of enzymatic digestion with Liberase (2.5 mg/ml, Roche) and DNaseI (10 mg/ml, Roche) diluted in PBS (Sigma) at 37°C. After filtration through a 100 µm cell strainer and resuspension in FACS buffer (PBS supplemented with 2% FBS (Sigma)), cell number was determined using a CASY cell counter (Innovatis). For most analyses CD45+ hematopoietic cells were depleted by incubation with anti-CD45 beads (Miltenyi) as per manufacturer`s recommendations and subsequently subjected to the AutoMACS separator (Miltenyi) "depleteS" program. Cells were counted and stained in FACS buffer containing antibodies of interest for 30 min at 4°C in the dark. For intra-cellular staining, cells were fixed and permeabilised after cell-surface staining using the Cytofix/Cytoperm (BD Biosciences) or the Fix/Perm buffer set (Invitrogen) according to the manufacturer`s protocol.
Instrument	BD LSRFortessa and BD FACSAria III were used for analysis and sorting.
Software	FACS DIVA (BD Biosciences) for acquisition and FlowJo v10 (TreeStar) for analysis.

Cell population abundance	All FACS sorted populations were sorted to a purity of 95% or greater.
Gating strategy	Samples were gated on size by FSC-A/SSC-A and doublets were excluded using SSC-W/SSC-H. Dead cells were removed by propidium iodide, 7AAD, or Zombie-red stain. Please see the gating strategy provided with the manuscript for subsequent gatings.

☒ Tick this box to confirm that a figure exemplifying the gating strategy is provided in the Supplementary Information.
